# Proteomics of early zebrafish embryos

**DOI:** 10.1186/1471-213X-6-1

**Published:** 2006-01-13

**Authors:** Vinzenz Link, Andrej Shevchenko, Carl-Philipp Heisenberg

**Affiliations:** 1 Max Planck Institute of Molecular Cell Biology and Genetics, Pfotenhauer Str. 108, 01307 Dresden, Germany

## Abstract

**Background:**

Zebrafish (*D. rerio*) has become a powerful and widely used model system for the analysis of vertebrate embryogenesis and organ development. While genetic methods are readily available in zebrafish, protocols for two dimensional (2D) gel electrophoresis and proteomics have yet to be developed.

**Results:**

As a prerequisite to carry out proteomic experiments with early zebrafish embryos, we developed a method to efficiently remove the yolk from large batches of embryos. This method enabled high resolution 2D gel electrophoresis and improved Western blotting considerably. Here, we provide detailed protocols for proteomics in zebrafish from sample preparation to mass spectrometry (MS), including a comparison of databases for MS identification of zebrafish proteins.

**Conclusion:**

The provided protocols for proteomic analysis of early embryos enable research to be taken in novel directions in embryogenesis.

## Background

The zebrafish has become a widely used vertebrate model system for which a large tool-box of genetic and cell biological methods has been established [1,2]. Research using zebrafish is further supported by the zebrafish sequencing project, which has facilitated the generation of microarrays for large scale expression profiling. It has been proposed that proteomics should complement the genome-wide expression profiling [3]. However, a major obstacle in the application of proteomics has been the high proportion of yolk proteins in early embryos. Proteomic studies in zebrafish have therefore been limited to adult tissues [4]. One study targeted larval stages 48 or 72 hpf (hours post fertilization), when the yolk to cell mass ratio is already decreased [5], however, without identifying the proteins. Therefore, it remains unclear whether at this stage analysis without deyolking provides satisfactory information about cellular proteins. Thus, the development of a reliable method to remove the interfering yolk from cells on a large scale is required to apply proteomics to early embryos.

Here, we provide detailed protocols for all zebrafish-specific steps of a proteomic experiment from dechorionation to mass spectrometry-based protein identification. As a key step, we present and validate a method for batch removal of the yolk from early embryos.

## Results

### Deyolking of embryos

**Figure 1:**
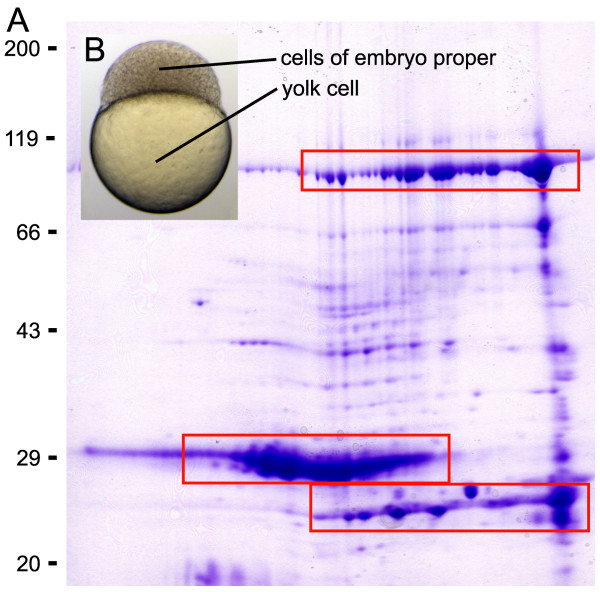
**The bulk of total protein in the early embryo is yolk protein**. **A**. Coomassie blue stained 2D gel (pI 3–10) of 1 mg protein extracted from shield stage embryos (6 hpf) without prior removal of the yolk. Several isoforms and degradation products of the predominant yolk protein Vitellogenin were spread over large parts of the gel (three boxes). Vitellogenin was identified by mass spectrometry. **B**. Embryo at high stage (3 1/3 hpf). The volume of the yolk cell exceeds the volume of the cells constituting the embryo proper.

In the early embryo, the cells forming the embryo proper constitute only a minor volume of the embryo compared to the large yolk cell (Fig. 1B). The abundance of yolk proteins interferes with any proteomic application that intends to target the cells of the embryo proper. The major yolk protein Vitellogenin, a phospholipo-glycoprotein, functions as a nutritional source for the developing embryo [6]. Figure 1A demonstrates how several isoforms and degradation products of Vitellogenin obscure the 2D gel image completely.

To generate enhanced 2D gels from early embryos, we developed a method for rapid batch removal of the yolk. The method takes advantage of the high mechanical instability of the big yolk cell compared to the smaller cells of the embryo proper. By pipetting with a narrow tip, the yolk cell can be disrupted. A buffer of low osmolarity facilitated the dissolving of the yolk. The deyolking efficiency was further increased by two additional wash steps.

**Figure 2:**
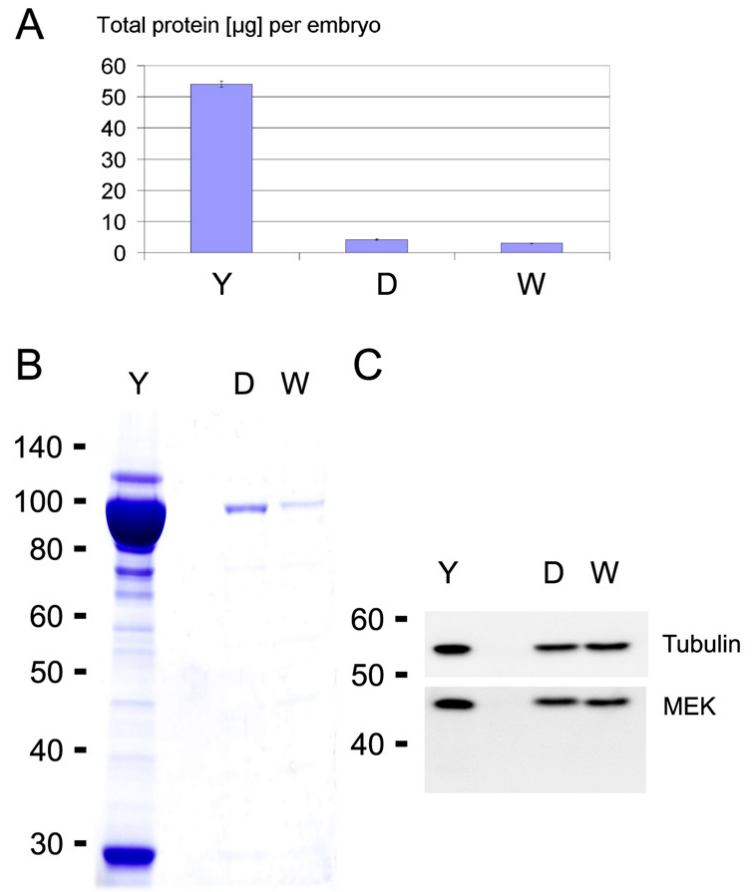
**Efficiency of yolk removal**. Embryos with yolk (Y) were analysed in comparison with embryos after one-step deyolking (D) or after two additional wash steps (W). **A**. Total protein amount per embryo as determined by DC protein assay (Bio-Rad). **B**. Coomassie stain (0.5 embryos loaded per lane). **C**. Western blot against Tubulin and MEK (0.5 embryos loaded per lane). While yolk proteins were efficiently depleted, recovery of cellular proteins remained high as evident by the minor reduction in signal intensity of Tubulin and MEK.

By removing the yolk proteins this method efficiently decreased the total protein amount per embryo more than 10 fold from 55 to 3 μg per embryo (Fig. 2A and 2B). However, recovery of cellular proteins remained high as evident by only a minor reduction in signal intensity of Tubulin and MEK as detected by Western blotting (Fig. 2C). We assume that this minor decrease is due to the loss of the fraction of MEK and Tubulin that is expressed in the yolk and yolk syncytial layer (YSL). There were no major changes in the efficiency of deyolking or the protein amount per embryo between high (3 1/3 hpf) and tailbud (10 hpf) stages.

### Western blotting

The success of Western blotting depends on the affinity and specificity of the antibodies used and on the abundance of the target protein. If the yolk is not removed manually, then only 1 or 2 embryos (50–100 μg) can be loaded per lane on a gel to avoid overloading effects due to yolk protein. This limits the sensitivity for cellular proteins. The deyolking method enabled us to load significantly more embryos and therefore the signal from specific cellular proteins was increased.

**Figure 3:**
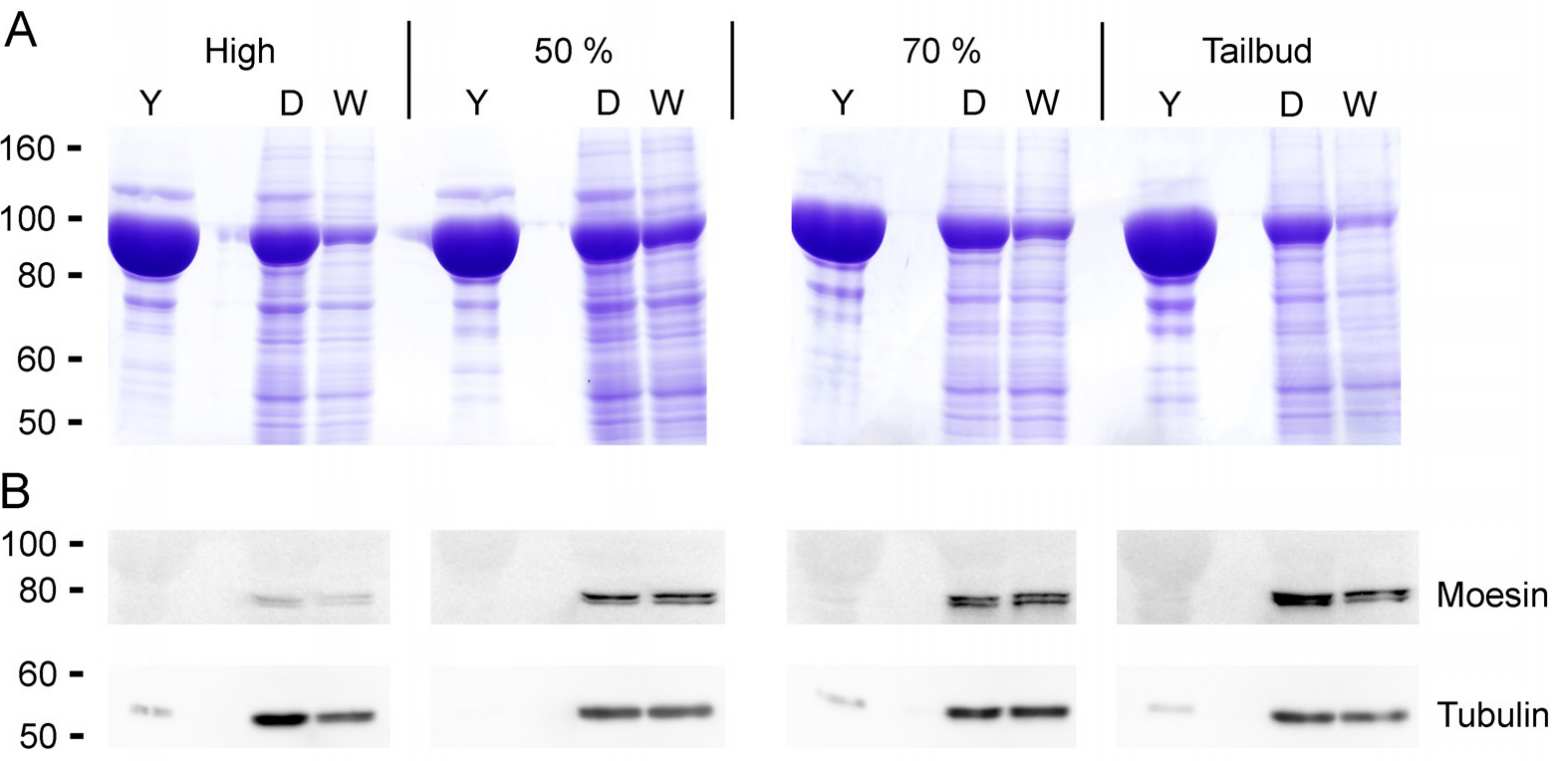
**Improved Western blotting results**. Embryos at high stage (3 1/3 hpf), 50% epiboly (5 1/4 hpf), 70% epiboly (7 hpf) and tailbud stage (10 hpf) were deyolked, separated by SDS-gel electrophoresis and Coomassie stained (A) or blotted and immunodetected with antibodies against Tubulin (55 kD) and Moesin (78/80 kD apparent molecular weight) (B). Note that total protein amount was lower in deyolked samples, therefore more embryos could be loaded per lane: 1 embryo with yolk (Y), 15 embryos deyolked (D), 15 embryos deyolked and washed twice (W). Consequently, signal intensities of cellular proteins were increased.

Figure 3 demonstrates the efficiency of the deyolking protocol and its benefits for Western blotting at four developmental stages from high (3 1/3 hpf) to tailbud (10 hpf) stage. For each stage one embryo with yolk was compared to 15 deyolked embryos by total protein staining (Coomassie) or specific detection (Western blotting) after SDS-gel separation. The Coomassie staining demonstrates that, despite loading 15 times more embryos, the major yolk protein band was clearly reduced after deyolking. The additional wash steps further reduced the level of yolk proteins (Fig. 3A). On the other hand, due to loading more embryos, the level of cellular proteins was significantly increased, as demonstrated by Western blotting: the part of the blot probed with Tubulin antibody shows a clear increase in band intensities. The naturally less abundant proteins of the Ezrin/Radixin/Moesin family fall below the detection limit if embryos were not deyolked, while after deyolking strong specific signals were observed starting at 50% epiboly (Fig. 3B).

The failure to detect Ezrin/Radixin/Moesin without deyolking may be due to electrophoretic distortions caused by abundant yolk sac proteins. Loading only 1 embryo (~50 μg protein), the dominant yolk protein induced major distortions in the molecular weight range of 80 to 100 kD. This decreased resolution and consequently also sensitivity around this mass range. Furthermore, the total protein amount that can be loaded without overloading artefacts is reduced in samples containing few, very abundant proteins compared to samples with uniformly distributed proteins, as after deyolking (Fig. 3A). We assume therefore that the increase in sensitivity gained due to this deyolking method even exceeds what would be anticipated due to the 10 fold reduction in total protein.

### 2D gel electrophoresis

**Figure 4:**
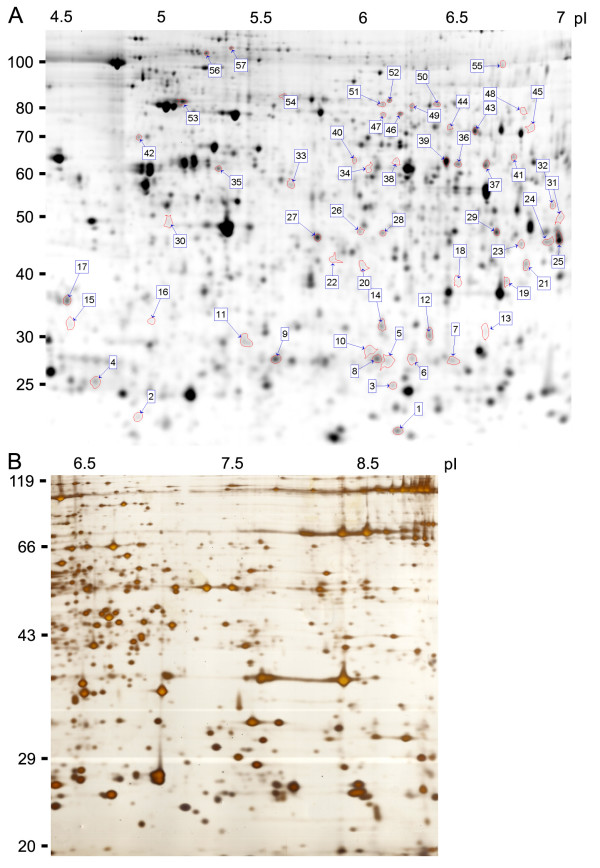
**2D gels**. 2D gel electrophoresis of samples derived from embryos at 80% epiboly (8 hpf) in the acidic as well as in the basic range. **A**. pI 4–7 (24 cm), fluorescently labelled with Cy2, 50 μg protein/dye. Spots processed for MS-identification are labelled (ID of Additional file 1). **B**. pI 6–9 (18 cm), silver stain.

The establishment of the deyolking protocol was a prerequisite for high quality 2D gels from early zebrafish embryos. After removal of the predominant yolk proteins we were able to generate high resolution 2D gels in the acidic (pI 4–7, Fig. 4A) as well as in the basic range (pI 6–9, Fig. 4B). We established a protocol that is compatible with three colour fluorescent labelling using the Ettan DIGE system (Amersham Biosciences), which significantly reduces inter-gel variability compared to one colour stains [7] while the sensitivity is comparable to silver staining with a detection limit of less than 1 ng protein [8].

### Mass spectrometry

Identification of proteins by mass spectrometry, using conventional search algorithms, depends on the representation of the sequence (or a very close homolog) in the searched database. Since the genome sequencing project of zebrafish is still ongoing, sequence coverage in the databases is incomplete. We therefore compared three databases in regard to the success rate in protein identification from zebrafish samples. The number of zebrafish entries in Ensembl, TIGR, MSDB and NCBInr database is presented in Table 1. MSDB and NCBInr are two general databases commonly used for MS database search. The zebrafish peptide database of Ensembl is generated by automatic annotation of sequences generated by the zebrafish genome project [9]. The TIGR gene index integrates EST-sequencing data from international zebrafish gene research projects and assembles single ESTs to "Tentative Consensus" sequences (TC) [10,11].

**Table 1:** Comparison of the number of zebrafish entries in databases applicable for MS identification

**Database**	**Release**	**Zebrafish Entries**
MSDB	20050515	12,168
NCBInr	20050627	14,505
Ensembl gene predictions	31.4d	23,524
TIGR	16.0	93,442

**Figure 5:**
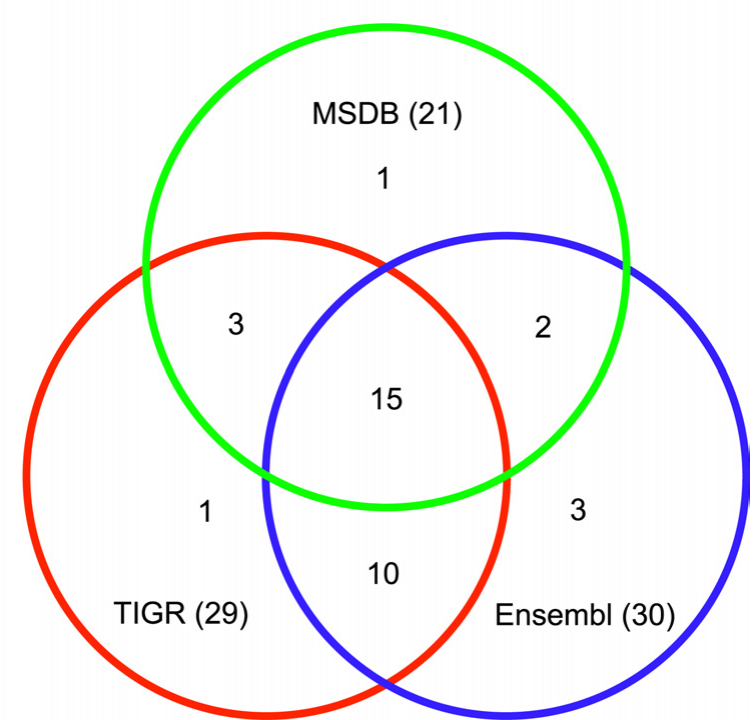
**Comparison of database performance for MS-identification**. Spots were analysed by MALDI-TOF and subsequently searched against the MSDB, Ensembl and TIGR database. The diagram depicts the number of validated positive identifications by the respective databases and the overlap between the databases. Using MSDB and Ensembl database all but one of the 35 unique proteins could be identified.

We compared the identification rate of these databases by analysing 57 out of 1400 spots automatically detected on a 2D gel. Besides a few higher expressed proteins, we selected many spots in the medium expression range or even close to the detection limit for MS-identification (Fig. 4A). In-gel tryptic digests of protein spots were analysed by MALDI-TOF and the peptide mass fingerprints (PMF) were subsequently searched against Ensembl, TIGR and MSDB databases [see Additional file 1]. All identifications were manually validated taking into account the MOWSE score, apparent versus predicted isoelectric point and molecular weight, as well as the error distribution of apparent versus predicted peptide masses (*m/z*). In total, 51 out of 57 spots were successfully identified. Figure 5 demonstrates how the identifications are distributed among the databases. 16 proteins that were identified from several spots were included only once in the statistics to avoid a bias. None of the databases alone was sufficient to identify all 35 unique spots. Using Ensembl and TIGR databases 30 and 29 spots could be identified, respectively, while the lower number of zebrafish entries in the MSDB database was reflected by a lower identification rate (21 proteins). The combination of two databases was sufficient for more than 95% of the identifications. We therefore routinely perform searches on MSDB and Ensembl databases. The TIGR database serves as an additional resource for samples which otherwise cannot be identified despite high quality spectra.

Both the Ensembl as well as the TIGR project gather a rich pool of information regarding the database entries, such as protein name, homologies, links to gene information and protein-domains. These valuable resources can be accessed directly by the identifiers of the respective proteins.

## Discussion

Our development of an efficient deyolking protocol for early embryos will influence many areas of zebrafish research. Most remarkably, it enabled us to undertake comparative proteomics of zebrafish embryos, an approach that has already proven its value in many other systems. In particular, the application of the DIGE system will support sensitive and quantitative analysis of protein expression differences. In addition to gel-based proteomics, the deyolking protocol also is crucial for gel free mass spectrometry-based proteomics.

There are numerous applications for quantitative proteomics in zebrafish: analysis of specific zebrafish mutants as well as the analysis of gain and loss of functions induced by RNA or Morpholino injections will provide a better molecular understanding of genes and their functions during development. Also, chemical inhibitors could be applied to elucidate downstream targets of specific signalling pathways. Finally, in the field of toxicology, proteomics will complement existing techniques like microarray analysis to reveal the mode of action of toxic substances. This will be increasingly important given that zebrafish as a model system in toxicology is on the rise [12].

In addition to its application in proteomics deyolking will be beneficial to other subjects such as microarray experiments targeting the gene expression pattern in the embryo proper. We have already applied the protocol successfully to such an experiment (data not shown). Therefore, the deyolking protocol will prove a valuable tool in zebrafish research including manifold applications in biochemistry and proteomics as well as other areas.

## Conclusion

We have established methods for proteomics of early zebrafish embryos. The simple but efficient protocol established for yolk removal enabled the generation of high quality 2D gels. It should further prove valuable for several other applications for which the yolk is detrimental. Here, we demonstrated that Western blotting is improved significantly using the deyolking method. The evaluation of mass spectrometry-based database searches revealed that the combination of two publicly available databases yields a good identification rate. Therefore, as a fruit of the zebrafish genome project, mass spectrometry-based identification of zebrafish samples is now possible. We hope that the developed methods will facilitate and stimulate the application of proteomics in zebrafish embryos to analyse fundamental developmental processes.

## Methods

### Dechorionation

Prior to generation of samples for Western blotting or 2D gel electrophoresis the chorion should be removed. We followed the method of Westerfield [1] to partially digest the chorion with Pronase so that the embryos fall out of their chorions. A heated (37°C) solution of 2 mg/ml Pronase (Roche Applied Science) in E2 (15 mM NaCl, 0.5 mM KCl, 2.7 mM CaCl_2_, 1 mM MgSO_4_, 0.7 mM NaHCO_3_, 0.15 mM KH_2_PO_4_, 0.05 mM Na_2_HPO_4_) was applied on the embryos. Since dechorionated embryos younger than tailbud stage adhere to plastic, dechorionation and subsequent steps should be performed on agar coated dishes (2% agar in E2). In our hands, extended Pronase incubation times of 15 to 30 min did not harm the embryo and facilitated the chorion removal, while the embryo was often squeezed and damaged if incubation time was too short. Several hundred embryos could easily be dechorionated in a 35 mm diameter Petri dish. After dechorionation, Pronase has to be completely removed before electrophoresis since otherwise remaining protease activity leads to a degradation of the high molecular weight proteins during the SDS gel run. Removal may be achieved, either by washing the embryos several times with E2, or by additional washing steps after deyolking.

### Deyolking embryos in batches

The principle behind the following method is to disrupt the yolk sac by mechanical stress. Subsequently, the yolk is dissolved in an appropriate buffer so that it stays in the supernatant during low speed centrifugation.

Embryos were transferred from the agar coated dish to a 1.5 ml tube filled with 1 ml deyolking buffer (1/2 Ginzburg Fish Ringer [1] without Calcium: 55 mM NaCl, 1.8 mM KCl, 1.25 mM NaHCO_3_) by pipetting with a narrow tip so that the yolk sac is disrupted (200 μl tip, Sarstedt 70.760.502). Up to 100 embryos can be transferred in a 200 μl volume. The embryos were shaken for 5 min at 1100 rpm to dissolve the yolk (Thermomixer, Eppendorf). Cells were pelleted at 300 g for 30 sec and the supernatant discarded. Care was taken not to disrupt the soft cell pellet. Optionally two additional wash steps were performed by adding 1 ml of wash buffer (110 mM NaCl, 3.5 mM KCl, 2.7 mM CaCl_2_, 10 mM Tris/Cl pH8.5), shaking 2 min at 1100 rpm and pelletting the cells as before. The additional wash steps further decreased the yolk and are recommended for 2D gel electrophoresis. The samples were frozen in liquid nitrogen or processed directly for Western blotting or 2D gel electrophoresis.

#### Optional modifications

Deyolking may be performed at 4°C to slow down cellular processes. For standard applications it performed well at room temperature.

Due to the lack of calcium the cells dissociate during the deyolking. Addition of 2.7 mM CaCl_2 _to the deyolking buffer preserves bigger cell clusters or cell sheets. Deyolking was only slightly less efficient in the presence of CaCl_2_.

### Western blotting

The samples were dissolved in 2 μl 2× SDS-sample buffer per embryo and incubated for 5 min at 95°C. No homogenisation was necessary since the cells dissolved rapidly in the buffer. After full speed centrifugation for 1 min in a microcentrifuge to remove insoluble particles, samples were loaded on a gel (per lane 10–15 embryos for a minigel, 15–30 embryos for large format gels). If not enough sample buffer was added, a slurry formed (presumably from DNA). If a semi-quantitative analysis is needed, care should be taken to load the same amounts of embryos in every lane. Since the volume of cells and remaining supernatant after deyolking is hard to control, we suggest to (1) either deyolk only the desired number of embryos and to load the whole sample or to (2) deyolk a sufficiently large number of embryos so that the volume of cells and remaining supernatant can be neglected against the large volume of added sample buffer.

Electrophoresis, blotting and detection was performed essentially as described in [13]. Briefly, 10% SDS-gels (10 × 10 cm) were run, semi-dry-blotted onto PVDF-membrane as described in the manual (Immobilon P, Millipore), stained for 5 min with Ponceau S, blocked 1 h in 5% nonfat dry milk in PBST (0.5% Tween-20 in PBS), incubated over night at 4°C with primary antibody in blocking buffer, washed 30 min with 4 changes of PBST, incubated 1 h with secondary antibody in blocking buffer and washed 30 min with 4 changes of PBST. Membranes were incubated for 5 min in ECL Plus (Amersham Biosciences) and emitted light was detected using a cooled CCD-camera (LAS-1000, Fujifilm).

#### Antibodies and dilutions used

MEK1/2: Cell Signaling #9122, 1:1000

alpha-Tubulin: Sigma T6199, 1:2000

Moesin: BD Biosciences 610401, 1:1000

### 2D gel electrophoresis

Based on the procedures for 2D gel electrophoresis described in [14], we optimized conditions and settings specifically for the zebrafish samples. After the last washing step of the deyolking procedure, care was taken to remove the supernatant completely in order to keep the salt concentration as low as possible. Total protein precipitation, to reduce salts and other interfering substances, is not required for analytical gels but may be applied for preparative gels. We recommend as starting material 50–100 embryos for analytical gels and 500 embryos for preparative gels.

### Methanol-chloroform precipitation

Lyse sample in 150 μl 3% SDS solution for 5 min at 90°C (for more than 250 embryos add multiples of 150 μl and divide into separate tubes after lysis); add 600 μl MeOH; add 150 μl chloroform, vortex (should give only one phase); add 450 μl water; vortex thoroughly; spin 2 min at 14,000 g; remove upper phase (without disturbing the interphase, which contains the proteins); add 450 μl MeOH; vortex thoroughly; spin 2 min; remove supernatant; air dry pellet 15 min.

### Sample preparation

To protect against protease activity, we added a mix of protease inhibitors (inhibitor mix) to solutions before the 1st dimension: 1 mM EDTA, 10 μM E64c, 0.002 mg/ml Aprotinin and 1 μM Pepstatin A (final concentrations).

Samples were lysed in rehydration buffer (pI 4–7: 7 M Urea, 2 M Thiourea, 4% Chaps, 5% Isopropanol, 2.5% Glycerol, 1% DTT, 5 μl/ml Bio-Lytes 3/10 (Bio-Rad), inhibitor mix) or in 30 μl lysis buffer (pI 6–9: 7 M Urea, 2 M Thiourea, 4% Chaps, 5 μl/ml IPG buffer pH 6–11 (Amersham Biosciences), 10 mM DTT, inhibitor mix). Samples were incubated with shaking for 15 min at 8°C after the addition of 0.5 μl Benzonase (25 U/μl > 99% purity, Novagen) to degrade DNA/RNA. Insoluble particles were removed by centrifugation for 1 h at 60,000 g. Protein concentration was determined by RC DC Protein assay (Bio-Rad). We loaded 150 μg protein on analytical gels and approximately 1 mg on preparative gels.

### DIGE

For DIGE labelling (Amersham Biosciences), 20 μl of lysis buffer (7 M Urea, 2 M Thiourea, 4% Chaps, 30 mM Tris pH 9.2, inhibitor mix) was added on the cells of 100 deyolked embryos. The sample was incubated with shaking for 15 min at 8°C after the addition of 0.5 μl Benzonase (25 U/μl > 99% purity, Novagen) to degrade DNA/RNA. Insoluble particles were removed by centrifugation for 1 h at 60,000 g. Protein concentration was determined by RC DC Protein assay (Bio-Rad). 50 μg protein was then labelled with 200 pmol CyDye as described in the user manual (Amersham Biosciences). The samples labelled with different dyes were combined and mixed with rehydration buffer (pI 4–7: 7 M Urea, 2 M Thiourea, 4% Chaps, 5% Isopropanol, 2.5% Glycerol, 1% DTT, 5 μl/ml Bio-Lytes 3/10 (Bio-Rad), inhibitor mix) or brought to 10 mM DTT and 5 μl/ml IPG buffer pH 6–11 (Amersham Biosciences) for cup loading (pI 6–9).

### Strip loading

Rehydration loading was applied for strips of pI 4–7 with the recommended volumes, while strips of pI 6–9 were cup-loaded in a volume of 20 – 50 μl after rehydrating the strips over night with rehydration buffer for basic strips (7 M Urea, 2 M Thiourea, 4% Chaps, 5 μl/ml IPG buffer pH 6–11 (Amersham Biosciences), 12 μl/ml Destreak (Amersham Biosciences), inhibitor mix).

### IEF conditions

1^st ^dimension isoelectric focusing was performed on Protean IEF cell (Bio-Rad):

pI 4–7 strips 24 cm (Amersham Biosciences): 30 min linear 0 V to 150 V, 1.5 h 150 V, 1 h 250 V, 4 h linear 250 V to 1000 V, 1.5 h linear 1000 V to 5000 V, 2 h linear 5000 V to 10,000 V, 10,000 V for 80 kVhrs, 500 V till proceeding to 2^nd ^dimension. pI 6–9 strips 18 cm (Amersham Biosciences): 2 h 150 V, 3 h 300 V, 4 h linear 300 V to 10,000 V, 10,000 V for 105 kVhrs, 500 V till proceeding to 2^nd ^dimension.

### 2nd dimension

Strips were reduced (20 mg/ml DTT in equilibration buffer: 6 M Urea, 2% SDS, 30 % glycerol, 0.375 M Tris pH8.8, 0.002 % bromphenol blue) and alkylated (25 mg/ml Iodoacetamide in equilibration buffer) 15 min each as described [15] and loaded on 10% SDS-gels with an overlay of agarose solution. Gels of figures 1A and 4B were run with Protean II xi cell (Bio-Rad) (2 h 5 mA/gel, > 12 h 75 V), the gel of figure 4A was run on Ettan Daltsix (Amersham Biosciences) (2 h 8 mA/gel, 16 h 85 V).

Silver staining was performed as described [16]. DIGE labelled gels were scanned with Typhoon 9410 Variable Mode Imager (Amersham Biosciences).

### Mass spectrometry

#### In-gel digestion

Protein spots were excised manually with a scalpel following silver or colloidal Coomassie staining [17]. Gel pieces were cut to cubes of approximately 1 × 1 × 1 mm length, reduced, alkylated and in-gel digested with trypsin as described previously [16]: after washing with water, water was removed and gel pieces were shrunk by dehydration with 50 μl acetonitrile for 15 min. Acetonitrile was removed and the proteins were reduced with 50 μl 10 mM dithiothreitol in 100 mM NH_4_HCO_3 _for 30 min at 56°C. The solution was removed, gel pieces were dehydrated as before and alkylated with 55 mM iodoacetamide in 100 mM NH4HCO3 for 20 min at room temperature in the dark, followed by a washing step (15 min) with 200 μl of 100 mM NH_4_HCO_3 _and dehydration as before. Gel pieces were then rehydrated for 2 h on ice in a 0.5 μM solution of bovine trypsin (sequencing grade, Roche, Mannheim, Germany) in 50 mM NH_4_HCO_3 _with the minimal volume sufficient to cover the pieces after rehydration (usually 10–20 μl). Samples were digested overnight at 37°C.

#### MALDI-TOF analysis

A 1.2 μl aliquot of the digestion solution was pipetted onto an AnchorChipTM 384/600 target (Bruker Daltonics GmbH, Bremen, Germany) as described [18]: 0.6 μl of matrix solution (2 mg α-Cyano-4-hydroxycinnamic acid [CHCA, Bruker Daltonik GmbH] dissolved in 0.33 ml of 2.5% aqueous TFA and 0.66 ml acetonitrile) was spiked directly into the analyte droplet. The mixture was allowed to dry at room temperature (approximately 30 min). Upon drying, the entire surface of the target was submerged in 5% formic acid for 2–3 min and then allowed to dry at room temperature for another 15–30 min. MALDI-TOF spectra in the *m/z *range 830–3000 were acquired on a Reflex IV (Bruker Daltonik, Bremen, Germany).

#### Database search

The obtained peptide mass fingerprints were searched with MASCOT 1.8 (Matrix Science LTD, U.K.) against the following publicly available databases:

NCBInr [19]

MSDB [20]

Ensembl zebrafish peptide database [21]

TIGR zebrafish gene index [22]

#### MASCOT search parameters

+/- 150 ppm peptide mass tolerance, enzyme specificity: trypsin, 1 missed cleavage tolerated, fixed modifications: carbamidomethyl

Variable modifications: MSDB, Ensembl and TIGR were searched with no variable modification. MSDB and Ensembl were in addition searched considering the following variable modifications: oxidation (histidine, tryptophan, methionine), double oxidation (tryptophan)

Proteins were considered as identified if the MOWSE score exceeded the threshold score (p = 0.05) for false positive hits.

#### Link to Ensembl and TIGR online resources

Both Ensembl and TIGR support direct access to the extended online information:





Where <identifier> has to be substituted by the respective identifier (the first word of each fasta-database entry).

## Authors' contributions

VL designed and performed the experiments and drafted the manuscript. CPH and AS conceived the experiments and revised the manuscript. All authors read and approved the final manuscript.

## Supplementary Material

Additional File 1 Protein identifications. 1 Unique spot identifier (ID) relating to figure 4A. 2 Molecular weight (MW) in kD and isoelectric point (pI) as apparent from the position on the 2D gel. 3 Databases which succeeded in identification are marked by "X". Only the first identification of each protein was included in the statistics. Disregarded identifications are marked by "(X)". 4 Description of protein function or name of homologous proteins. 5 Basis for the description: TIGR Description as assigned by TIGR zfin Protein/gene name assigned by zfin. Vega Protein/gene name assigned by Vega. NCBI Protein/gene name reported at NCBI REFSEQ Protein/gene name from Refseq. BLAST close homologue based on BLAST-search. Ensembl characterizes their orthologue predictions into UBRH (U)nique (B)est (R)eciprocal (H)it. MBRH one of (M)ultiple (B)est (R)eciprocal (H)its. RHS (R)eciprocal (H)it based on (S)ynteny around BRH. HS human, MM mouse, CE *C. elegans*. 6 MW and pI predicted from the sequence. 7 MOWSE score/threshold score (p < 0.05 of false positives for scores higher than threshold). 8 Peptides matched to identified protein/fingerprint masses searched. 9 Sequence coverage of the matched peptides. 10 (R)oot (M)ean (S)quare error in ppm of predicted versus apparent peptide mass (*m/z*). 11 Database. 12 Corresponding accession number of identified proteins. 13 Link to sequences and online resources (Ensembl and TIGR only). Click here for file
